# Supplementary material for: Analysis of Half a Billion Datapoints Across Ten Machine-Learning Algorithms Identifies Key Elements Associated With Insulin Transcription in Human Pancreatic Islet Cells
Source: Front Endocrinol (Lausanne). 2022 Mar 23;13:853863. doi: 10.3389/fendo.2022.853863 (PMC8986156; doi:10.3389/fendo.2022.853863)
Supplement: Supplementary file 1 [file DataSheet_1.docx]

# **Supplementary information:**

**Detailed methods**

***Pancreatic single-cell (sc)RNA sequencing datasets and analyses.***

Analysis was carried out by using R studio version 1.2.5033, the SeuratData (version 0.2.1) and Seurat (3.2.3). Data normalization across multiple datasets in Panc8 are described previously [1]. Single-cell selection criteria involved all cells that contained transcript data (samples with all zeros eliminated). Single-cell datasets were randomly split to 80% samples selected in a training set with the remaining 20% retained for validation. ML-based identification of features predicting insulin transcript was carried out by data scientists blinded to gene variables and sample information. Analytical plans (80/20 discovery and validation) were predetermined and gene identifiers/sample details were provided once models were ranked following in silico validation.

**Supplementary Table 1.**

| **#** | **Workflow** | **Build** | **Description** |
| --- | --- | --- | --- |
| **1** | **Random Forest** | Ensemble | Random Forest classifier is a meta-estimator that fits several decision trees on various sub-samples of datasets and uses an average to improve the predictive accuracy of the model and controls over-fitting. The sub-sample size is always the same as the original input sample size, but the samples are drawn with replacement. Reduction in over-fitting and Random Forest classifier is more accurate than decision trees in most cases. |
|  |  |  |  |
| **2** | **Gradient Boosting** | Ensemble | The idea behind "gradient boosting" is to take a weak hypothesis or weak learning algorithm and make a series of tweaks to it that will improve the strength of the hypothesis/learner. This type of Hypothesis Boosting is based on the idea of Probability Approximately Correct Learning (PAC). |
|  |  |  |  |
| **3** | **Adaptive Boosting (Adaboost)** | Ensemble | For AdaBoost, many weak learners are created by initializing many decision tree algorithms that only have a single split. The instances/observations in the training set are weighted by the algorithm, and more weight is assigned to instances that are difficult to classify. More weak learners are added into the system sequentially, and they are assigned to the most difficult training instances. In AdaBoost, the predictions are made through majority vote, with the instances being classified according to which class receives the most votes from the weak learners. |
|  |  |  |  |
| **4** | **Ridge Classifier** | Regularization | Ridge classifier first converts binary targets to [-1, 1] and then treats the problem as a regression task, optimizing the same objective as above. The predicted class corresponds to the sign of the regressor’s prediction. For multiclass classification, the problem is treated as multi-output regression, and the predicted class corresponds to the output with the highest value. |
|  |  |  |  |
| **5** | **Logistic Regression** | Regression | Logistic regression is a machine learning algorithm for classification. In this algorithm, the probabilities describing the possible outcomes of a single trial are modelled using a logistic function. It is most useful for understanding the influence of several independent variables on a single outcome variable. |
|  |  |  |  |
| **6** | **Naive Bayes** | Bayesian | Naive Bayes algorithm based on Bayes’ theorem with the assumption of independence between every pair of features. Naive Bayes classifiers work well in many real-world situations such as document classification and spam filtering. This algorithm requires a small amount of training data to estimate the necessary parameters. Naive Bayes classifiers are extremely fast compared to more sophisticated methods. |
|  |  |  |  |
| **7** | **Decision Tree Classifier** | Decision Tree | Given data of attributes together with its classes, a decision tree produces a sequence of rules that can be used to classify the data. Decision Tree is simple to understand and visualise, requires little data preparation, and can handle both numerical and categorical data. |
|  |  |  |  |
| **8** | **K-Nearest Neighbours** | Instance-based | Neighbours based classification is a type of lazy learning as it does not attempt to construct a general internal model but simply stores instances of the training data. Classification is computed from a simple majority vote of the k nearest neighbours of each point. This algorithm is simple to implement, robust to noisy training data, and effective if training data is large. |
|  |  |  |  |
| **9** | **Linear Discriminant Analysis**  **(LDA)** | Dimensionality Reduction | It is a classifier with a linear decision boundary, generated by fitting class conditional densities to the data and using Bayes’ rule. The model fits a Gaussian density to each class, assuming that all classes share the same covariance matrix. The fitted model can also be used to reduce the dimensionality of the input by projecting it to the most discriminative directions, using the transform method. |
|  |  |  |  |
| **10** | **Linear Support Vector Classifier** | Support Vector Machines (SVM) | Support vector machine is a representation of the training data as points in space separated into categories by a clear gap that is as wide as possible. New examples are then mapped into that same space and predicted to belong to a category based on which side of the gap they fall. |

**Supplementary Table 1:** Description of the ten different ML workflows used for predictive analyses. Related to Figure 2A.

**Supplementary Table 2.**

| Rank | Gradient Boost | Random Forest | ADA Boost | Decision tree |
| --- | --- | --- | --- | --- |
| 1 | IAPP | IAPP | IAPP | MTRNR2L8 |
| 2 | MTRNR2L8 | ADCYAP1 | SST | IAPP |
| 3 | MTRNR2L1 | MAFA | ATP5E | MAFA |
| 4 | RPS15 | SST | LDHA | GSTA1 |
| 5 | SST | DLK1 | ADCYAP1 | RPS15 |
| 6 | MAFA | PCSK1N | GPX4 | MAB21L3 |
| 7 | ADCYAP1 | HADH | DOCK10 | XIST |
| 8 | NPTX2 | PCSK1 | CAPN13 | CRP |
| 9 | LDHA | MALAT1 | KCNA5 | RPL35 |
| 10 | MAB21L3 | LDHA | RPL23AP32 | SST |
| 11 | PCSK1N | NPTX2 | NPM3 | NPTX2 |
| 12 | DDX3Y | CD99 | GGPS1 | CNPY3 |
| 13 | MIF | C1QL1 | RHOBTB1 | PCSK1N |
| 14 | RBP4 | EEF1A2 | SIX3 | TP73-AS1 |
| 15 | ATP5E | CD151 | RPL13AP17 | CCDC28A |
| 16 | RPS28 | GNAS | STMN3 | IGSF3 |
| 17 | CRP | RBP4 | DDX6 | RPL26 |
| 18 | GSTA1 | MIF | PLSCR4 | HADH |
| 19 | MAST1 | EEF2 | FAM163A | MZT2B |
| 20 | CD24 | UCHL1 | ZNF578 | RPL36A |
| 21 | RPS18 | IGF2 | MTRNR2L8 | MIR663A |
| 22 | AES | LRRFIP1 | RPL27A | MARK2 |
| 23 | PRSS8 | HNRPDL | CST3 | ZNF682 |
| 24 | TRIM47 | UGDH-AS1 | PRRC2C | C6orf1 |
| 25 | CD151 | MTRNR2L2 | MTRNR2L6 | BNIP2 |
| 26 | GPX4 | RPS17 | KCTD21 | EEF1A2 |
| 27 | HADH | MAB21L3 | ARPC3 | KIAA1161 |
| 28 | ATP5MD | TMEM66 | KCTD1 | TGFBI |
| 29 | MYO6 | RPL3 | LINC00342 | RPS4Y1 |
| 30 | L1TD1 | ITGB1 | ADSSL1 | AHSA2 |
| 31 | TTTY15 | ABHD2 | DLK1 | RNFT2 |
| 32 | XIST | ERO1LB | STRN | NFASC |
| 33 | CST3 | ANXA4 | SNHG14 | PHC2 |
| 34 | LOC101929224 | TTR | MYL6 | CCNG2 |
| 35 | DLK1 | ABCC8 | LINC00294 | BMS1 |
| 36 | RPL8 | YWHAZ | FXYD3 | RSBN1 |
| 37 | LINC01550 | GPX4 | WSCD2 | SDF2L1 |
| 38 | C4BPB | ATP5E | HIST1H1E | LINC01858 |
| 39 | MTRNR2L10 | CD59 | CACNA2D3 | FTH1 |
| 40 | PRRC2C | HNRNPU | DCTN4 | FBXL18 |
| 41 | UBL7-AS1 | KCNQ1OT1 | MAFA | EIF1B |
| 42 | GNAS | MTRNR2L1 | H1FX | BBX |
| 43 | ATAD2 | PRRC2C | TTTY15 | TAF7 |
| 44 | AL353803.1 | WSCD2 | ANO6 | FXYD6 |
| 45 | HSPA6 | TOMM6 | SUPT20H | IRF2BP2 |
| 46 | UCHL1 | NLRP1 | S100A14 | NMB |
| 47 | LOC100287177 | UGGT1 | LYPD3 | TMA7 |
| 48 | THSD7A | SRRM2 | SCARF1 | KLF7 |
| 49 | LINC00342 | RPS15 | SMC3 | RPL17 |
| 50 | RPS4Y1 | CPE | MAST1 | KIAA1919 |
| 51 | GGPS1 | CCAR1 | CCDC38 | SRPR |
| 52 | GCG | SCGN | ZNF790-AS1 | GNG5 |
| 53 | ZFP36L1 | H3F3A | MARS | RASGRF1 |
| 54 | BRWD3 | PFKFB2 | ZNF787 | CXCL3 |
| 55 | SACS | ANKRD12 | AKAP1 | ZPBP |
| 56 | IVNS1ABP | WAC-AS1 | UGT2B17 | ADCYAP1 |
| 57 | ITGB1 | LPP | CA5BP1 | PNMA3 |
| 58 | BBS12 | GCG | TRAPPC12 | PPP1CB |
| 59 | PCDHA8 | C17orf76-AS1 | PRPF19 | ALDOA |
| 60 | EDA | RPS18 | RAB30-AS1 | CHGA |
| 61 | GAD2 | ZC3HAV1 | RDH13 | C2CD2L |
| 62 | BRI3 | TMSB4X | HADH | DOT1L |
| 63 | PRSS3P2 | RPL36A-HNRNPH2 | DENND6A | IGIP |
| 64 | RPS29 | RPL8 | RPS29 | HERPUD1 |
| 65 | TP73-AS1 | RHOC | ATAD2 | TMEM254 |
| 66 | MTHFD1 | REG3A | SLC16A3 | EDARADD |
| 67 | GOLIM4 | STX16 | RPS15 | GADD45B |
| 68 | AC025254.1 | YWHAB | DROSHA | FOXA3 |
| 69 | PTBP2 | SRSF11 | WBP11 | SLC35B4 |
| 70 | SERINC1 | RNASEK | SEZ6 | ABHD5 |
| 71 | KCNJ2 | PKD1P1 | SNORA79 | MALL |
| 72 | PSAP | PEMT | FAM83H | LOC101928069 |
| 73 | EHF | MYO10 | MTCH2 | RPS20 |
| 74 | AKR7A2 | FTL | FAM114A1 | SRP9 |
| 75 | PKD1P1 | C1orf127 | PITPNA | A1BG |
| 76 | AC025259.3 | LINC00657 | LHPP | DDAH1 |
| 77 | MYOF | RPL13AP20 | BRPF3 | C7orf65 |
| 78 | PRPF19 | ERO1B | IFITM1 | TRIM33 |
| 79 | MIR663A | PTPRN | BSG | ARHGAP35 |
| 80 | YPEL3 | SCG5 | CSDE1 | YOD1 |
| 81 | EIF3J-DT | MYO6 | MTHFD1 | LRRC10 |
| 82 | AC098934.2 | PPIA | WBP1 | PIGK |
| 83 | 38596 | TM4SF1 | EHBP1 | SERTM1 |
| 84 | CPEB3 | FXYD2 | C4BPB | SERPINB6 |
| 85 | LOC100506476 | U2AF1 | NEURL3 | ING2 |
| 86 | CENPC1 | PTP4A2 | EFNB3 | GLTSCR2 |
| 87 | BAI2 | PPP1R1A | RBP4 | SURF1 |
| 88 | UEVLD | LOC643406 | LINC00657 | LOC100507463 |
| 89 | MX1 | GAD2 | SPNS1 | CYP2R1 |
| 90 | ARID4B | FBLIM1 |  | LGALS9 |
| 91 | MDFI | SOD2 |  | GDF15 |
| 92 | FLJ33534 | RPS14 |  | SLC35B3 |
| 93 | ATP6V0C | TMSB10 |  | TXNIP |
| 94 | RAB30-AS1 | SYT13 |  | ERCC-00004 |
| 95 | CREG1 | RPSAP58 |  | C1orf56 |
| 96 | RPL3 | B2M |  | EIF3L |
| 97 | ATP6V0B | SYT7 |  | RCAN3 |
| 98 | AKIP1 | RPL24 |  | AGMO |
| 99 | TENM2 | RPS28 |  | MRP63 |
| 100 | HYPK | TIMP2 |  | PCDHB2 |

**Supplementary Table 2:** List of up to top 100 genes ranked by their importance in association with presence of insulin transcript in a single cell, identified from the four selected ML methods (Gradient Boosting; Random Forest; ADA Boost; Decision Tree). Related to Figure 3D.

**Supplementary Table 3.**

| **#** | **37 common elements in "AdaBoost", "DecTree", "GraBoost" and "RanForest":** |
| --- | --- |
| 1 | amylin receptor signaling pathway (GO:0097647) |
| 2 | calcitonin family receptor signaling pathway (GO:0097646) |
| 3 | cardiolipin acyl-chain remodeling (GO:0035965) |
| 4 | cotranslational protein targeting to membrane (GO:0006613) |
| 5 | establishment of protein localization to endoplasmic reticulum (GO:0072599) |
| 6 | fatty acid beta-oxidation (GO:0006635) |
| 7 | histamine secretion (GO:0001821) |
| 8 | histamine transport (GO:0051608) |
| 9 | hormone-mediated apoptotic signaling pathway (GO:0008628) |
| 10 | insulin secretion (GO:0030073) |
| 11 | mRNA catabolic process (GO:0006402) |
| 12 | negative regulation of acute inflammatory response (GO:0002674) |
| 13 | negative regulation of acute inflammatory response to antigenic stimulus (GO:0002865) |
| 14 | negative regulation of acute inflammatory response to non-antigenic stimulus (GO:0002878) |
| 15 | negative regulation of amyloid fibril formation (GO:1905907) |
| 16 | nuclear-transcribed mRNA catabolic process (GO:0000956) |
| 17 | nuclear-transcribed mRNA catabolic process, nonsense-mediated decay (GO:0000184) |
| 18 | peptide hormone secretion (GO:0030072) |
| 19 | positive regulation of calcium ion import across plasma membrane (GO:1905665) |
| 20 | positive regulation of somatostatin secretion (GO:0090274) |
| 21 | protein targeting to ER (GO:0045047) |
| 22 | regulation of acute inflammatory response to non-antigenic stimulus (GO:0002877) |
| 23 | regulation of amyloid fibril formation (GO:1905906) |
| 24 | regulation of calcium ion import across plasma membrane (GO:1905664) |
| 25 | regulation of hormone levels (GO:0010817) |
| 26 | regulation of hormone secretion (GO:0046883) |
| 27 | regulation of oligodendrocyte progenitor proliferation (GO:0070445) |
| 28 | regulation of peptide hormone secretion (GO:0090276) |
| 29 | regulation of somatostatin secretion (GO:0090273) |
| 30 | response to extracellular stimulus (GO:0009991) |
| 31 | RNA catabolic process (GO:0006401) |
| 32 | sensory perception of pain (GO:0019233) |
| 33 | spermine biosynthetic process (GO:0006597) |
| 34 | spermine metabolic process (GO:0008215) |
| 35 | SRP-dependent cotranslational protein targeting to membrane (GO:0006614) |
| 36 | viral gene expression (GO:0019080) |
| 37 | viral transcription (GO:0019083) |

**Supplementary Table 3:** The 37 common pathways (elements) targeted by the top 10 features from each of the four selected ML methods (RF: Random Forest; GB: Gradient Boosting; ADAB: ADA Boost; DT: Decision Tree) identified using gene ontology (GO) function analysis. Related to Figure 3D. List ordered in alphabetical order.

**Supplementary Table 4**

| **Gene transcript** | **P-value** | **ND** | **T2D** |
| --- | --- | --- | --- |
| *INS* | <0.0001 | 9.31 ± 0.29 | 6.52 ± 0.19 |
| *IAPP* | <0.0001 | 6.88 ± 0.28 | 4.56 ± 0.20 |
| *ADCYAP1* | <0.0001 | 3.24 ± 0.24 | 1.81 ± 0.16 |
| *MAFA* | 0.0021 | 0.89 ± 0.10 | 0.71 ± 0.08 |
| *SST* | <0.0001 | 5.23 ± 0.25 | 3.02 ± 0.16 |
| *LDHA* | 0.2679 | 6.67 ± 0.26 | 6.85 ± 0.19 |

**Supplementary Table 4:** The key genes common in all top three ML workflows (Figure 3C) with *INS* gene accessed in single-cell RNA-seq data (GSE154126 [2]) between adult non-diabetic (ND; N=296) vs T2D (N=505) insulin transcribing single cells. Data are presented as log_2_ transformed normalized reads mean + SEM. The P-values were calculated with the Mann-Whitney test. Genes with significant P-value (P<0.05) show here, remain significant after adjusting for multiple testing using the Benjamini-Hochberg method (FDR=0.05, on 21,153 tests).


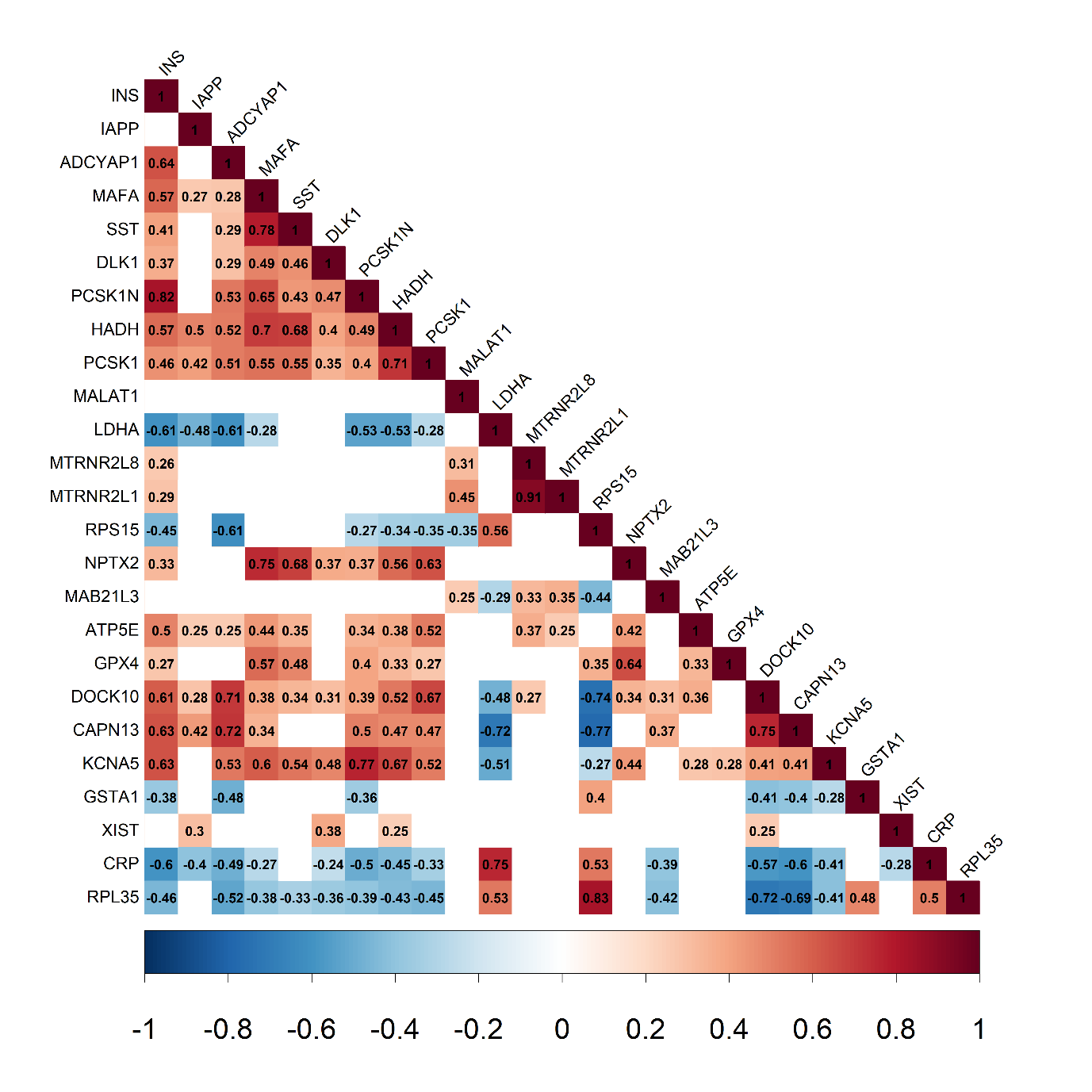


**Supplementary Figure 1:** **Selected genes validated in human islet bulk-RNA-seq dataset.**

Spearman correlation matrix of the top gene transcripts selected in the four ML algorithms (as presented in Figure 3C) along with insulin transcript on the human islet bulk RNA-seq dataset (GSE152111; n=66). RPL23AP32 was not present in the dataset. Only significant correlation (Spearman p-value < 0.05) are presented with colour along with the Spearman correlation r coefficient in black text within the square. Positive correlation is shown in shades of red, while negative correlation is in shades of blue.

**References**

[1] T. Stuart, A. Butler, P. Hoffman, C. Hafemeister, E. Papalexi, W.M. Mauck, 3rd, Y. Hao, M. Stoeckius, P. Smibert, and R. Satija, Comprehensive Integration of Single-Cell Data. Cell 177 (2019) 1888-1902 e21.

[2] D. Avrahami, Y.J. Wang, J. Schug, E. Feleke, L. Gao, C. Liu, H. Consortium, A. Naji, B. Glaser, and K.H. Kaestner, Single-cell transcriptomics of human islet ontogeny defines the molecular basis of beta-cell dedifferentiation in T2D. Mol Metab 42 (2020) 101057.
